# EGF and EGFR Facilitate Alveolar Development by Promoting the Proliferation of Alveolar Type II Cells in the Yak (*Bos grunniens*)

**DOI:** 10.3390/cells15131167

**Published:** 2026-06-26

**Authors:** Biao Wang, Xiaowen Zhang, Yan Cui, Junfeng He, Sijiu Yu, Qian Zhang, Shijie Li, Huizhu Zhang

**Affiliations:** 1 Laboratory of Animal Anatomy & Tissue Embryology, Department of Basic Veterinary Medicine, Faculty of Veterinary Medicine, Gansu Agricultural University, Lanzhou 730070, China; wangb0503@163.com (B.W.); 18293553355@163.com (X.Z.); hejf@gsau.edu.cn (J.H.); sijiuy@126.com (S.Y.); zq880204@126.com (Q.Z.); 107331901031@st.gsau.edu.cn (S.L.); 2 Gansu Province Livestock Embryo Engineering Research Center, Department of Clinical Veterinary Medicine, Faculty of Veterinary Medicine, Gansu Agricultural University, Lanzhou 730070, China; 3 College of Animal & Veterinary Sciences, Southwest Minzu University, Chengdu 610041, China; lanbel0831@163.com

**Keywords:** yak, AT2 cells, proliferation, apoptosis, alveolar development

## Abstract

**Highlights:**

**What are the main findings?**

- Primary alveolar type II epithelial cells from yaks were successfully isolated and cultured, marking a significant step in the study of their respiratory biology.
- Activation of the EGF/EGFR signaling pathway significantly enhances the proliferation of yak AT2 cells and promotes their cell cycle progression via the AKT and STAT3 pathways.

**What are the implications of the main findings?**

- The EGF/EGFR signaling pathway plays a pivotal role in fostering alveolar development in yaks, enabling them to thrive in the challenging conditions of high altitude and low oxygen environments.

**Abstract:**

Yaks (*Bos grunniens*) are large mammals endemic to the Qinghai–Tibet Plateau. Efficient lung development is crucial for their adaptation to high-altitude hypoxia. As progenitor cells of the alveoli, type II alveolar epithelial (AT2) cells warrant further investigation into their physiological functions; however, relevant studies remain limited. In this study, primary AT2 cells were isolated from the lungs of yaks. Concurrently, lung tissues were collected from yaks at distinct developmental stages to investigate the role of the EGF/EGFR axis in regulating AT2 cell proliferation and apoptosis, as well as its essential contribution to yak lung development. Here, we demonstrate that the EGF/EGFR axis plays a beneficial role in yak alveolar development. Exogenous EGF supplementation or EGFR activation upregulated the downstream factors AKT and STAT3, enhanced AT2 cell proliferation, and reduced apoptosis. In contrast, EGFR inhibition promoted AT2 cell apoptosis and suppressed proliferation. Cell cycle analysis revealed that both exogenous EGF and EGFR activation increased the proportion of AT2 cells in the S and G2 phases, whereas EGFR inhibition caused cell cycle arrest at the G0/G1 phase. Moreover, the expression of cell cycle regulators cyclin D1, CDK4, and CDK6 was upregulated, while p16 and p21 expression was downregulated. Further comparative analyses indicated that the EGF/EGFR axis positively contributes to alveolar development in juvenile yaks. Collectively, these findings confirm that in plateau environments, activation of the EGF/EGFR axis promotes AT2 cell proliferation and inhibits apoptosis, thereby facilitating alveolar development in juvenile yaks. A key limitation is the lack of parallel comparisons with low-altitude cattle and other plateau-endemic species (e.g., Tibetan sheep), which precludes definitive assessment of the specificity of the EGFR/EGF axis in yak AT2 cell proliferation and lung development.

## 1. Introduction

The yak, a ruminant inhabiting high-altitude regions at an average elevation of over 3000 m, has exhibited enhanced adaptability to the plateau environment through prolonged genetic evolution. The alveoli, serving as a crucial site for gas exchange, encompass two distinct types of epithelial cells: type 1 (AT1) and type 2 (AT2). While AT1 cells play a pivotal role in maintaining the blood–gas barrier [1], they exhibit limited proliferative capacity and heightened susceptibility to oxygen-induced damage. AT2 cells are regional pulmonary epithelial progenitor cells that proliferate and generate alveolar structures during lung development [2]. After birth, the alveoli undergo a series of remodeling processes and enter the alveolar stage. In humans, alveolarization is a postnatal process characterized by an increase in the number of alveoli from approximately 50 million at birth to over 300 million in adulthood, highlighting the crucial role of tightly regulated rapid proliferation of AT2 cells after birth for subsequent alveolar development [3,4]. Yaks inhabit high-altitude environments with low oxygen levels for extended periods, so the rapid development of alveoli plays a pivotal role in facilitating efficient oxygen exchange within their organisms [5,6]. Given the pivotal role of AT2 cells in respiratory physiology, their precise quantity and proliferation are indispensable during normal lung development in yaks, as well as during the reparative process following exposure to hypoxia.

Numerous studies have demonstrated the intricate nature of signaling pathways involved in promoting AT2 cell proliferation. For instance, keratinocyte growth factor (KGF) and hepatocyte growth factor (HGF) have been identified as mitogens for rat AT2 cells in vitro [7,8]. In mouse models, fibroblast growth factor (FGF) signaling is crucial for alveolar cell fate selection [9]. Among these pathways, the epidermal growth factor receptor (EGFR) plays a pivotal role. As a transmembrane tyrosine kinase receptor, EGFR is implicated in regulating cell cycle progression across diverse cell types [10,11,12]. Using EGFR knockout mouse models, it has been demonstrated that loss of EGFR leads to aberrant alveolar development and respiratory difficulties [13,14], highlighting its non-redundant role in lung morphogenesis. Furthermore, studies on human and rodent lung epithelial cells have shown that EGF can stimulate AT2 cell proliferation by activating EGFR and may be involved in regulating alveolar epithelial repair processes [15].

Nevertheless, the precise function of EGFR in yak lung AT2 cell proliferation and alveolar development remains elusive. As typical plateau indigenous mammals, yaks form unique pulmonary structural adaptations to long-term hypoxic stimulation. Existing yak-focused studies have confirmed that the critical window of postnatal alveolar development is 30–180 days after birth, during which alveolar quantity and volume increase sharply, and the blood–gas barrier gradually thins to improve gas exchange efficiency under low oxygen [6]. When lowland mammals are transferred to high altitude, hypobaric hypoxia induces sequential alveolar lesions. Long-term plateau exposure triggers irreversible injuries, including diffuse interstitial fibrosis, alveolar simplification and surfactant dysfunction [16]. Previous studies have shown that under hypoxic conditions, the EGFR/PI3K/AKT signaling axis can alleviate inflammatory responses, oxidative stress, and restore mitochondrial function, thereby alleviating lung injury induced by high-altitude hypoxia [17]. Following EGFR activation, it can initiate pivotal downstream signaling cascades, including the PI3K-AKT pathway and the JAK/STAT signaling pathway; however, the activation of downstream signals exhibits species and cell specificity [18,19,20]. Furthermore, studies using mouse models have confirmed that blocking EGFR can promote apoptosis of alveolar epithelial cells and inhibit the repair of lung injury [21]. As for the role of this signal axis in the development of the yak’s lungs, there are currently no relevant reports available.

From this, it can be inferred that EGFR may serve as a pivotal regulatory molecule and play a crucial role in maintaining normal respiratory function in yaks. Therefore, further investigation is warranted to elucidate the specific involvement of EGFR in AT2 cell proliferation and lung development in yaks, thereby unraveling its precise regulatory mechanism underlying healthy respiratory system function in yaks.

## 2. Materials and Methods

### 2.1. Animals and Sample Collection

Lung tissues were obtained from healthy domesticated yaks at various developmental stages: newborns (0~3 days, *n* = 3), juveniles (6 months–1 year, *n* = 3), adults (2~3 years, *n* = 3), and seniors (7~8 years, *n* = 3), in Xining, Qinghai Province. One part of the tissue samples was preserved in a 4% paraformaldehyde (PFA) solution for histological examination, while the other part was promptly frozen in liquid nitrogen for molecular biological studies. This research has received approval from the Animal Ethics Committee at Gansu Agricultural University (Approval Numbers: GSAU-Eth-VMC-2025-010).

### 2.2. Isolation and Culture of Yak AT2 Cells

Fresh lung tissue from 3-day-old yaks was rinsed with saline, and alveolar tissues were dissected free of blood vessels, airways, and connective tissue. The tissue was digested with 0.25% trypsin (27250018, Gibco, Carlsbad, CA, USA) and 0.1% type I collagenase (17100017, Gibco, Carlsbad, CA, USA) at 37 °C for 60 min. The cell suspension was filtered through a 100-mesh sieve and centrifuged at 1500 rpm. Two successive purification steps were performed to eliminate fibroblast and AT1 cell contamination: (1) differential adherence purification: cell suspension was seeded in culture flasks for 1 h, fibroblasts rapidly adhered to the bottom, and unattached suspended AT2 cells were collected to remove fibroblast impurities; (2) differential digestion purification: 0.05% trypsin was used for short-term digestion to strip adherent fibroblasts, while tightly aggregated AT2 cell colonies remained intact. Cells at passages 2–3 with an over 95% SPC-positive rate (detected by immunofluorescence) were used for subsequent functional experiments to exclude AT1 cell contamination.

All subsequent in vitro cell experiments were carried out under hypoxic conditions (5% O_2_) to recapitulate the high-altitude environment in which yaks naturally reside.

### 2.3. Transmission Electron Microscopy (TEM)

For transmission electron microscopy, AT2 cells were fixed in 3% glutaraldehyde and postfixed in 1% osmium tetroxide. After dehydration, the samples were embedded in EPON^®^ 812 resin. Semithin sections were stained with methylene blue, and ultrathin sections were counterstained with uranyl acetate and lead citrate. Specimens were then examined using a JEM-1400-FLASH transmission electron microscope (JEOL, Tokyo, Japan).

### 2.4. Immunofluorescence Staining

Lung tissues were fixed in 4% paraformaldehyde for 48 h, dehydrated, and embedded in paraffin; 4 μm sections were prepared. AT2 cells were fixed in 4% paraformaldehyde for 30 min, permeabilized with 0.5% Triton X-100, and blocked with 5% BSA (SW3015, Solarbio, Beijing, China). Primary antibodies against SPC (AP53886PU-N, OriGene, Rockville, MD, USA), Aqp5 (NBP3-12286, Novus Biologicals, Centennial, CO, USA), EGFR (bs-10007R, Bioss, Beijing, China), EGF (bs2010R; Bioss, Beijing, China), and Ki67 (GTX00538, Genetex, San Antonio, TX, USA) were incubated overnight at 4 °C. After washing, cells were incubated with Alexa Fluor 594-conjugated secondary antibody for 1 h at 37 °C, stained with DAPI, and imaged by fluorescence microscopy.

### 2.5. Cell Counting Kit-8 (CCK-8) Assay Cytotoxicity

Yak AT2 cells were seeded at 5000 cells/well in 96-well plates. After 24 h incubation, cells were starved for 12 h and treated with EGF, AG1478, or NSC228155 at specified concentrations. CCK-8 solution was added to each well and incubated for 3 h at 37 °C. Absorbance was measured at 490 nm using a microplate reader.

### 2.6. Quantitative Real-Time PCR (qRT-PCR)

Total RNA extracted with TRIzol (Solarbio, Beijing, China) was reverse-transcribed into cDNA using the EVO M-MLV Reverse Transcription Kit (Accurate Biology, Changsha, China). The cDNA was then amplified via qRT-PCR in 20 μL reactions containing SYBR Green Mix and gene-specific primers (Table 1). Relative quantification was performed using the 2^−ΔΔCt^ method, and *β-actin* served as the reference control.

### 2.7. Western Blotting

Cells and tissues were lysed in RIPA buffer with PMSF at 4 °C for 1 h. Protein concentrations were determined using a BCA assay. Proteins were separated by SDS-PAGE, transferred to PVDF membranes, and blocked with 5% skim milk for 3 h. Membranes were incubated overnight at 4 °C with primary antibodies against Bcl-2 (AF6138, Affinity, Changzhou, China), EGFR (bs-10007R, Bioss, Beijing, China), EGF (bs2010R, Bioss, Beijing, China), PCNA (bs-2006R; Bioss, Beijing, China), Bax (bs-20386R; Bioss, Beijing, China), P-AKT1 (AF0832; Affinity, Changzhou, China), AKT1 (AF6260; Affinity, Changzhou, China), P-STAT3 (AF3293, Affinity, Changzhou, China), STAT3 (AF6293; Affinity, Changzhou, China) and β-actin antibody (Bioss, Beijing, China). After washing, membranes were incubated with HRP-conjugated secondary antibody for 1 h at room temperature. Signals were detected using ECL reagent and quantified with ImageJ 1.8.0.345.

### 2.8. Cell Cycle Distribution Analysis

Cells were fixed in 70% ethanol overnight at 4 °C, washed with PBS, treated with RNase, and stained with propidium iodide (100 μg/mL) for 30 min on ice. Cell cycle distribution was analyzed by flow cytometry (FACS Calibur, BD Biosciences, Milpitas, CA, USA).

### 2.9. Annexin V-FITC/PI Assay

AT2 cells were plated at 2.0 × 10^5^ cells/well in 6-well plates and treated with drugs for 48 h. Cells were collected, washed with PBS, and resuspended in binding buffer. After staining with Annexin V-APC and 7-AAD for 30 min in the dark, apoptosis was analyzed by flow cytometry.

### 2.10. 5-Ethynyl-20-Deoxyuridine (EdU) Assay

AT2 cells were plated in 6-well cell culture plates, and transfection was initiated once the confluence of cells reached 30–40%. After a 24 h incubation post-transfection, cells were subjected to the BeyoClick^TM^ EdU-488 cell proliferation assay (C0071S, Beyotime Biotechnology, Shanghai, China), as per the manufacturer’s protocol. Thereafter, images of the cells were captured using fluorescence microscopy.

### 2.11. Statistical Analysis

Data are reported as means ± SD. Intergroup differences were assessed via one-way ANOVA (SPSS 25.0), and statistical significance was inferred at *p* < 0.05, *p* < 0.01, and *p* < 0.001. Figures were generated using GraphPad Prism 8.0.

## 3. Results

### 3.1. Primary Culture, Identification of Yak AT2 Cells, and Expression of EGF and EGFR

**Figure 1:**
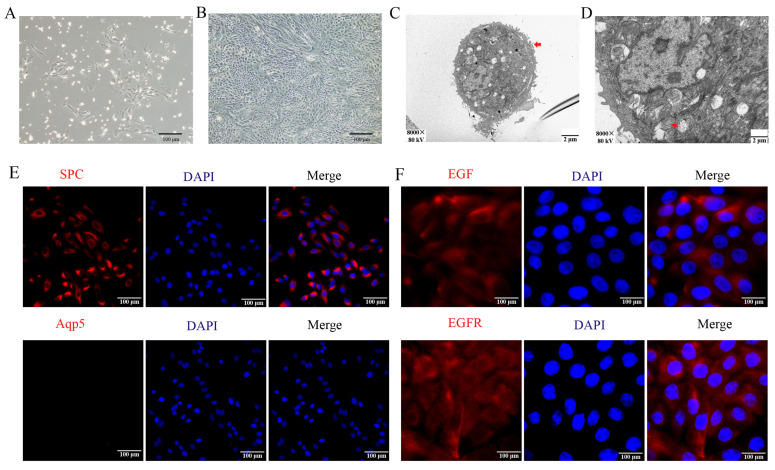
Isolation, culture, and characterization of yak primary AT2 cells. (**A**) Morphological characterization of primary yak AT2 cells following 72 h of in vitro cultivation. (**B**) The cultured AT2 cells exhibited a cobblestone-like morphology on the 6th day. (**C**,**D**) Identification of AT2 cells was performed using transmission electron microscopy. The thick red arrows indicate the microvilli on the surface of AT2 cells, while the thin red arrows indicate osmiophilic multilamellar bodies. (**E**) AT2 cells were identified through immunofluorescence detection, visualizing the presence of the AT2-marker protein SPC while simultaneously confirming the absence of the AT1-marker protein Aqp5. The nucleus was stained blue with DAPI. (**F**) The presence of EGF and EGFR in AT2 cells was ascertained through immunofluorescence staining. The nucleus was stained blue with DAPI.

Primary AT2 cells were successfully isolated and subsequently cultured from the pulmonary tissue of yaks. Within 72 h of initiating the culture, the cells adhered to and spread on the surface of the culture plate. After 6~7 days, they exhibited a confluent pavement-like morphology (Figure 1A,B). AT2 cells were purified through differential digestion and selective adherence. The designation of these cells as AT2 cells was validated by TEM analysis (Figure 1C). This identification was corroborated by the differential expression of cell-specific markers: SP-C for AT2 cells and AQP5 for AT1 cells (Figure 1E). These findings indicate that over 95% of the isolated cells were indeed identified as AT2. Furthermore, we investigated the presence of EGF and EGFR in primary yak AT2 cells. Our results demonstrated positive immunofluorescence signals for both EGF and EGFR in AT2 cells (Figure 1F).

### 3.2. Screening of the Optimal Treatment Concentration and Time for EGF, AG1478, and NSC228155

**Figure 2:**
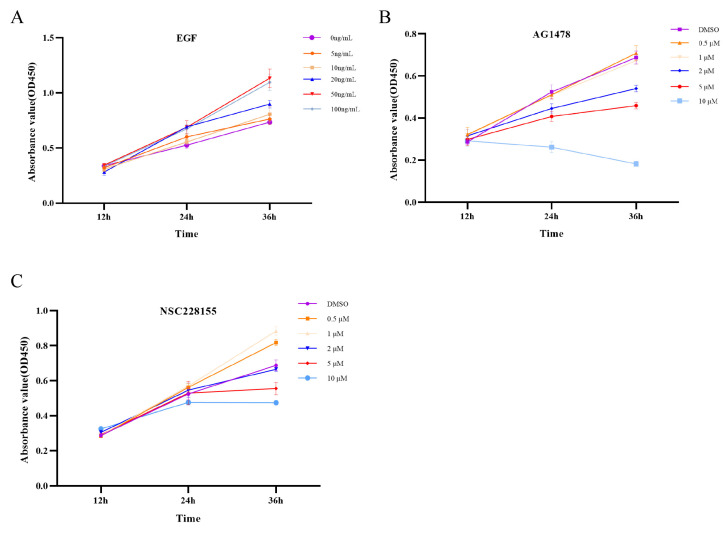
The concentration and duration of EGF, AG1478, and NSC228155 for optimal treatment screening. (**A**) AT2 cells were exposed to a range of EGF concentrations (0, 5, 10, 20, 50, and 100 mg/mL) and incubated for 12, 24, and 36 h. The cell viability was assessed by measuring the optical density (OD) at 450 nm using the CCK-8 assay (*n* = 4). (**B**) Similarly, AT2 cells were treated with varying concentrations of AG1478 (0, 0.5, 1, 2, 5, and 10 μM) over the same time intervals. The OD values, indicative of cell viability, were again determined using the CCK-8 assay (*n* = 4). (**C**) AT2 cells were also subjected to treatment with a series of NSC228155 concentrations (0, 0.5, 1, 2, 5, and 10 μM) for durations of 12, 24, and 36 h. The OD values were measured to evaluate cell viability, following the CCK-8 assay protocol (*n* = 4). Data are presented as means ± standard deviation (SD).

In order to investigate the effects of EGF and EGFR on yak AT2 cell proliferation, we supplemented the culture medium with EGF protein, as well as the EGFR inhibitor AG1478 and activator NSC228155. The optimal treatment time and potential cytotoxicity of these compounds on yak AT2 cells were determined using a CCK-8 assay. The absorbance results revealed that the optimal concentrations for EGF, AG1478, and NSC228155 were 50 ng/mL, 5 μM, and 1 μM respectively, with an ideal treatment duration of 36 h (Figure 2A–C). Consequently, these concentrations and treatment duration were employed in subsequent experiments.

### 3.3. EGF and EGFR Promote Proliferation and Inhibit Apoptosis in Yak AT2 Cells

**Figure 3:**
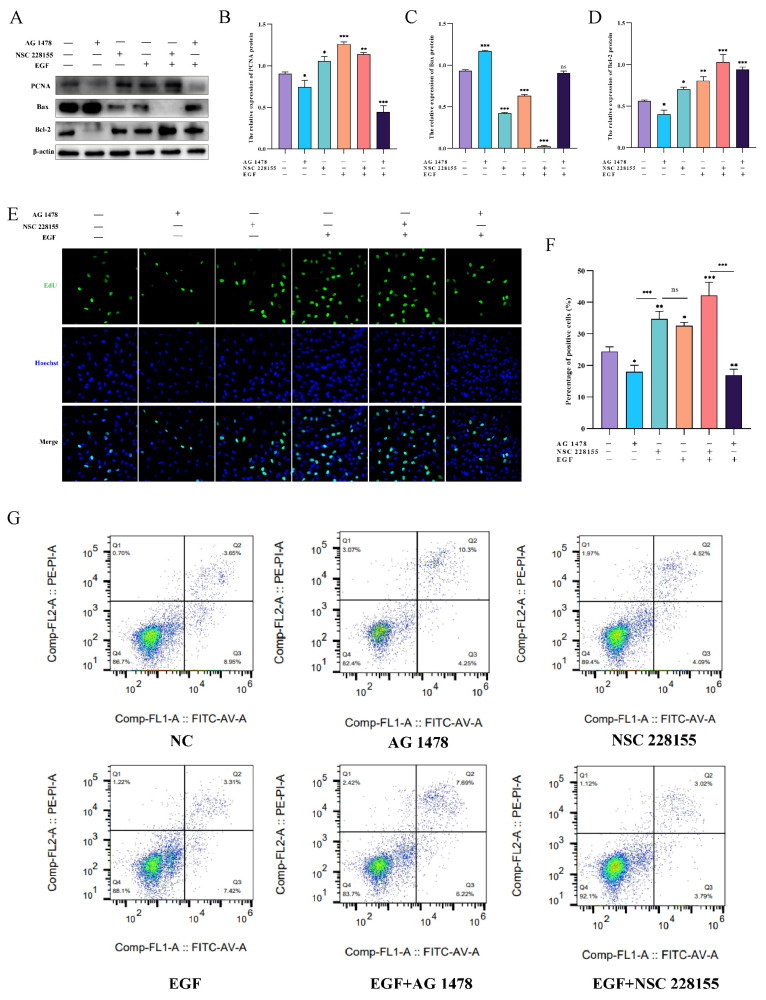
EGF and EGFR promote proliferation and inhibit apoptosis in yak AT2 cells. (**A**–**D**) The protein expression of PCNA, Bax, and Bcl-2 was assessed using Western blot following the addition of EGF, AG1478, and NSC228155 respectively. The gray value analysis was performed using ImageJ software (*n* = 3). (**E**,**F**) The number of positive cells was determined by EdU staining after treatment with EGF, AG1478, and NSC228155 respectively. Statistical analysis was conducted (*n* = 6). Magnification is 20×. Green indicates EdU-positive cells, while blue represents the cell nuclei. (**G**) Apoptosis rate was measured by flow cytometry. Data are presented as mean values ± standard deviation (SD). ‘ns’ denotes no significant difference, while asterisks indicate levels of statistical significance: * *p* < 0.05, ** *p* < 0.01, and *** *p* < 0.001.

To elucidate the effects of EGF and EGFR on the proliferation and apoptosis of AT2 cells in yaks, we conducted separate treatments with EGF protein, the EGFR inhibitor AG1478, and the EGFR activator NSC228155. Western blot analysis revealed that EGFR inhibition markedly reduced the expression of PCNA and Bcl-2 proteins, concurrently increasing the expression of Bax protein. In contrast, EGFR activation yielded the converse results. Furthermore, EGF supplementation significantly enhanced proliferation and attenuated apoptosis in yak AT2 cells (Figure 3A–D). Interestingly, simultaneous treatment with both EGF addition and EGFR activation resulted in a more pronounced increase in cell proliferation accompanied by a significant reduction in apoptosis. EdU staining showed that the proportion of EdU-positive cells was markedly decreased following EGFR inhibition, whereas addition of EGF factor, along with EGFR activation, significantly increased this proportion (Figure 3E,F). Flow cytometry data indicated that inhibiting EGFR led to an elevated rate of apoptosis in yak AT2 cells, while activating EGFR and adding EGF reduced this rate. In conclusion, these findings provide compelling evidence for the role played by EGF and EGFR in promoting cell proliferation while inhibiting apoptosis in yak AT2 cells.

### 3.4. Impact of EGF and EGFR on Downstream Factor Expression Within the EGFR Signaling Pathway

**Figure 4:**
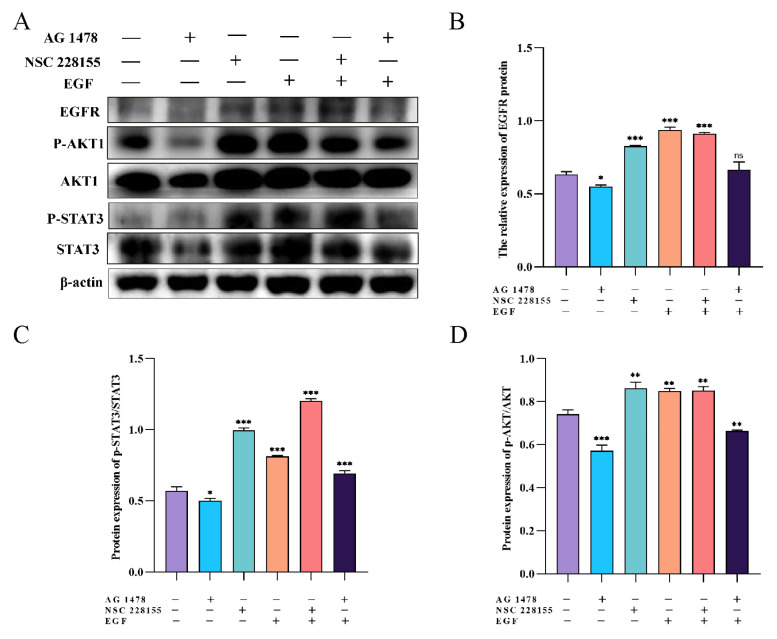
The influence of EGF, the EGFR inhibitor AG1478, and the EGFR activator NSC228155 on the expression profiles of downstream effectors within the EGFR signaling cascade. (**A**–**D**) Western blot was performed to detect the protein expression of EGFR, P-AKT1, AKT1, P-STAT3, and STAT3 after treatment with EGF, AG1478, and NSC228155 respectively. The resulting images were analyzed using ImageJ software to obtain gray values. (*n* = 3). Data are presented as mean values ± standard deviation (SD). ‘ns’ denotes no significant difference, while asterisks indicate levels of statistical significance: * *p* < 0.05, ** *p* < 0.01, and *** *p* < 0.001.

To identify the downstream signaling cascades by which EGF/EGFR promotes yak AT2 cell proliferation. We investigated the expression of downstream factors in the EGFR signaling pathway. Western blot analysis revealed that EGF stimulation and EGFR activation significantly enhanced the phosphorylation of AKT and STAT3 proteins, respectively. Conversely, inhibition of EGFR expression attenuated the phosphorylation levels of AKT and STAT3 proteins (Figure 4A–D). These findings suggest that EGFR promotes yak AT2 cell proliferation, potentially through both the EGFR-PI3K-AKT and EGFR-JAK/STAT3 pathways.

### 3.5. Effects of EGF and EGFR on the Cell Cycle in AT2 Cells of Yak

**Figure 5:**
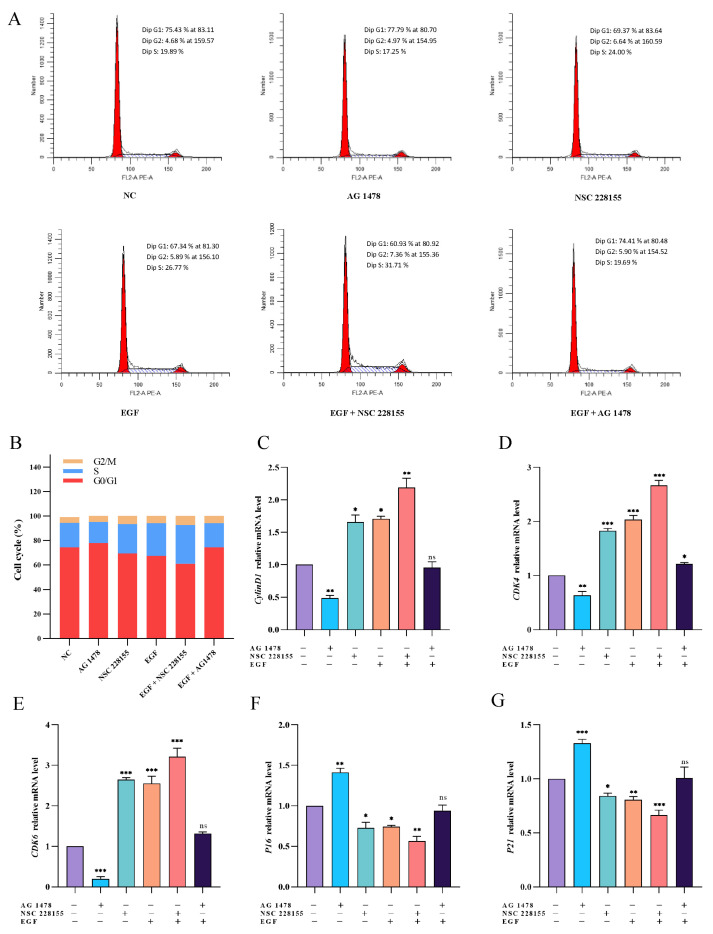
Effects of EGF and EGFR on the cell cycle in AT2 cells of Yak. (**A**,**B**) Cell cycle distribution was analyzed using flow cytometry (*n* = 3). (**C**–**G**) The relative expression levels of cell cycle-related genes *cyclin D1*, *CDK4*, *CDK6*, *p16* and *p21* mRNA were detected by qRT-PCR. (*n* = 4). Data are presented as mean values ± standard deviation (SD). ‘ns’ denotes no significant difference, while asterisks indicate levels of statistical significance: * *p* < 0.05, ** *p* < 0.01, and *** *p* < 0.001.

Given the integral relationship between cell proliferation and cell cycle progression, we utilized flow cytometry to analyze changes in the cell cycle distribution in response to EGF and EGFR stimulation in AT2 cells derived from yaks. As illustrated in Figure 5A,B, treatment with EGF, coupled with the addition of an EGFR activator, led to a significant increase in the number of cells entering the S and G2/M phases of the cell cycle. In contrast, the inclusion of EGFR inhibitors markedly impeded cell progression into these phases. Subsequently, we evaluated the mRNA expression levels of factors related to the cell cycle. qRT-PCR analysis indicated that EGF-treated cells, when supplemented with an EGFR activator, displayed a significant upregulation of *cyclin D1*, *CDK4*, and *CDK6* genes, which are pivotal for cell cycle progression. Conversely, the expression of cyclin-dependent kinase inhibitors *p16* and *p21* was notably reduced. Inhibition of EGFR activity resulted in the opposite effects (Figure 5C–G).

### 3.6. EGFR Signaling Pathway Facilitates Alveolar Development in the Yak

**Figure 6:**
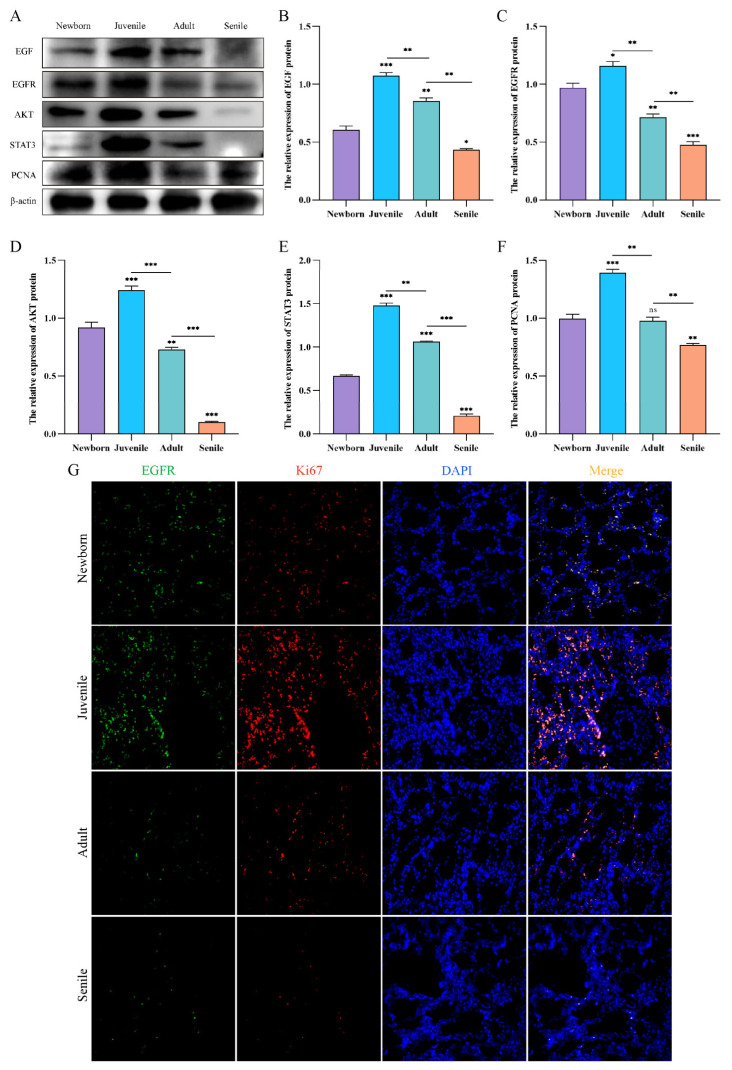
Effect of EGFR signaling pathway on alveolar development in yak. (**A**–**F**) Differences in the expression levels of EGF, EGFR, AKT, STAT3, and PCNA proteins in the lungs of newborn, juvenile, adult and senile yaks were detected by Western blot and analyzed as gray values using ImageJ software. (*n* = 3). (**G**) Immunofluorescence staining to detect the expression and distribution of EGFR and Ki67 in the lungs of newborn, juvenile, adult and senile yaks. Magnification is 20×. Green represents EGFR, red represents Ki67, and blue represents the cell nucleus. ‘ns’ denotes no significant difference, while asterisks indicate levels of statistical significance: * *p* < 0.05, ** *p* < 0.01, and *** *p* < 0.001.

To elucidate the role of the EGFR signaling pathway in the alveolar development of yaks, we assessed the expression profiles of EGFR and its downstream effectors across various stages of pulmonary maturation. Western blot revealed maximal PCNA expression in juveniles. This was accompanied by elevated levels of EGF, EGFR, AKT, and STAT3 proteins, which were significantly higher than those observed in all other developmental stages (Figure 6A–F). Conversely, these expressions were lowest in the senile group (Figure 6A–F). Subsequently, we confirmed the localization of EGFR and Ki67 expression during yak alveolar development using immunofluorescence staining. The results demonstrated that both EGFR and Ki67 cell positivity were highest in the juvenile group and lowest in the senile group (Figure 6G). These findings suggest a crucial involvement of the EGFR signaling pathway in yak alveolar development.

## 4. Discussion

This study not only elucidated the molecular mechanism underlying AT2 cell proliferation in yaks for the first time but also provided crucial insights for further investigation into alveolar development in this species. Our findings demonstrate that the EGF/EGFR/AKT and EGF/EGFR/STAT3-dependent pathways play a pivotal role in promoting AT2 cell proliferation and facilitating G1/S transition, while concurrently inhibiting AT2 cell apoptosis. Furthermore, we have substantiated that activation of the EGFR signaling pathway significantly contributes to alveolar development in yaks.

In this study, we present novel insights into the proliferation mechanism of AT2 cells and alveolar development in yaks, a large mammal adapted to prolonged exposure to low oxygen environments. We successfully cultured primary yak AT2 cells in vitro and observed a significant upregulation of EGF and EGFR expression in these cells. Subsequent activation and inhibition experiments demonstrated the active involvement of EGFR in yak AT2 cell proliferation. Studies have demonstrated that EGF/EGFR exerts a significant promotional effect on the proliferation of mouse AT2 cells [22]. EGFR participates in numerous metabolic processes, encompassing cell proliferation [23], differentiation [24], and oxidative stress response [25]. EGF and its cognate ligands, including transforming growth factor-α (TGF-α) and heparin-binding EGF-like growth factor (HB-EGF), engage with multiple EGFR/ErbB1, ErbB2/HER2, ErbB3/HER3, and ErbB4/HER4 receptor subtypes. These receptors are prominently expressed on the surface of pulmonary epithelial cells [26,27,28]. Upon ligand binding and subsequent autophosphorylation at several C-terminal tyrosine residues, the activated EGFR initiates intracellular signaling cascades by providing docking sites for various downstream signaling molecules [29]. The activation of EGFR induces mitosis and cell formation while also playing a crucial role in alveolar epithelial [30,31].

The progression of the cell cycle is intricately linked to the extent of cellular proliferation. In this study, it was observed that the addition of EGF or EGFR activators significantly augmented the proportion of cells entering the S-phase and G2/M-phase, while concomitantly reducing the fraction of cells residing in the G0/G1-phase. It has been demonstrated that cells arrested in G0 phase are referred to as quiescent cells, which retain their ability to re-enter a normal cell cycle and undergo well-ordered mitosis upon stimulation with mitogenic cues [32,33]. EGF/EGFR has the capacity to modulate the expression levels of cyclin D1 and p16 proteins; however, the precise downstream signaling pathways through which this regulation occurs remain unclear. Previous studies have reported that in mouse embryonic stem cells and primary cultured external auditory canal keratinocytes, the ERK and PI3K/AKT signaling pathways are associated with EGF-induced upregulation of cyclin D1 expression [34,35]. Additionally, in keratinocytes, matrix metalloproteinases mediate the EGFR/ERK/AKT/cyclin D1 signaling pathway to promote cell cycle progression from G1 phase to S phase [36,37]. These findings align with our experimental results on yak AT2 cells, which further confirm that EGF/EGFR and its downstream pathway facilitate cell proliferation.

The alveolar structure of the lung comprises two types of epithelial cells, namely AT2 and AT1. AT2 cells not only play a crucial role in producing lung surface-active substances but also serve as progenitors for AT1 cells by undergoing trans-differentiation into them. Given that AT1 cells lack proliferative capacity, the replenishment of these cells during alveolar development can solely be achieved through the proliferation and differentiation of AT2 cells themselves [38]. Consequently, the proliferation of AT2 cells plays a pivotal role in both yak lung development and repair processes associated with hypoxic lung injury. In this study, the expression levels of EGF/EGFR and downstream factor proteins in yak alveolar epithelial cells were found to be consistent with the degree of proliferation, reaching their highest levels during the juvenile period when proliferation was most pronounced. The critical period for yak lung development has been demonstrated to occur between 30 and 180 days after birth, during which significant alveolar development takes place. This is characterized by an increase in both the number and volume of alveoli, as well as a notable thinning of the air-blood barrier [6]. Previous studies have demonstrated that mice lacking the EGF/EGFR exhibit lung hypoplasia [14]. Given that yaks inhabit low-oxygen environments on plateaus for extended periods, their alveoli undergo rapid development to adapt to these conditions and ensure proper gas exchange within the body. Therefore, the observed high expression of EGF/EGFR in young yak lungs is likely attributed to their exposure to a hypoxic environment during critical periods of lung development between 30 and 180 days after birth. In conclusion, our findings suggest that EGF/EGFR promotes rapid alveolar development during the juvenile period in yaks.

A key limitation is the lack of parallel comparisons with low-altitude cattle and other plateau endemic species (e.g., Tibetan sheep, goats). Although plateau ungulates generally augment alveolar proliferation under hypoxic stress, upstream regulatory pathways are species-specific. Our data show that the EGF/EGFR–AKT/STAT3 axis drives AT2 proliferation and alveolar maturation in juvenile yaks, yet whether this represents a common Bovidae mechanism or a yak-specific trait remains unclear. Future isolation of AT2 cells from Tibetan sheep and yellow cattle, followed by comparative pathway activity assays under hypoxia, will elucidate their evolutionary adaptive features. Additionally, our current use of chemical inhibitors/activators precludes definitive causal conclusions due to the lack of gene-specific knockout. Upcoming work will employ whole-transcriptome RNA-seq to systematically identify EGF/EGFR-regulated downstream genes and novel hypoxia-adaptive signals, and CRISPR/Cas9-mediated single-gene knockdown (EGFR, AKT, STAT3) in yak AT2 cells to unravel their independent and cooperative functions in cell cycle/apoptosis under hypoxia, overcoming the limitations of pharmacological intervention.

## 5. Conclusions

In conclusion, these results indicate that EGF/EGFR signaling supports AT2 cell proliferation and alveolar remodeling during early yak lung development, a mechanism crucial for high-altitude respiratory adaptation. The absence of parallel comparisons with low-altitude cattle and other plateau-endemic species (e.g., Tibetan sheep) precludes assessment of EGFR/EGF axis specificity in yak AT2 proliferation and lung development, a gap that future work will fill.

## Figures and Tables

**Table 1:** Information on primers.

Genes	Primer Sequences (5′-3′)	Tm/°C
*Cylin-D1*	F: 5′-CGCCCTCGGTGTCCTACTTC-3′ R: 5′-CCTCGCAGACCTCCAGCAT-3′	60
*CDK4*	F: 5′-GTGTACAAGGCCCGTGATCC-3′ R: 5′-TGCACAGACGTCCATAAGCC-3′	58
*CDK6*	F: 5′-TGCACAGACGTCCATAAGCC-3′ R: 5′-CGTTAGTTTGGTTTCTCTGT-3′	58
*P16*	F: 5′-TGCGCCGGTTCTTGATTACA-3′ R: 5′-CCCATCATCATCACCTGGTCT-3′	60
*P21*	F: 5′-ACTTGGACCTGTCGCTGT-3′ R: 5′-GGAGTGGTAGAAATCTGTCAT-3′	58
*β-actin*	F: 5′-TCCTGCGGCATTCACGAAACTAC-3′ R: 5′-GTGTTGGCGTAGAGGTCCTTGC-3′	58

## Data Availability

The original contributions presented in this study are included in the article. Further inquiries can be directed to the corresponding author.

## Abbreviations

The following abbreviations are used in this manuscript:

AT2	Alveolar type 2
SP	Surfactant-associated protein
AT1	Alveolar type 1
HIF	Hypoxia-induced factor
EGF	Epidermal growth factor
EGFR	Epidermal growth factor receptor
PFA	Paraformaldehyde
PBS	Phosphate-buffered saline
DAPI	4′,6-diamidino-2-phenylindole
qPCR	Quantitative Real-Time PCR
TEM	Transmission electron microscopy
CCK-8	Cell Counting Kit-8
SEM	Standard error of the mean
ANOVA	Analysis of variance
H&E	Hematoxylin and eosin
KGF	Keratinocyte growth factor
HGF	Hepatocyte growth factor
